# FNG-IE: an improved graph-based method for keyword extraction from scholarly big-data

**DOI:** 10.7717/peerj-cs.389

**Published:** 2021-03-11

**Authors:** Noman Tahir, Muhammad Asif, Shahbaz Ahmad, Muhammad Sheraz Arshad Malik, Hanan Aljuaid, Muhammad Arif Butt, Mobashar Rehman

**Affiliations:** 1 Department of Computer Science, National Textile University, Faisalabad, Punjab, Pakistan; 2 Department of Information Technology, Government College University, Faisalabad, Faisalabad, Punjab, Pakistan; 3 Computer Sciences Department, College of Computer and Information Sciences, Princess Nourah bint Abdulrahman University (PNU), Riyadh, Sudia Arabia; 4 Punjab University College of Information Technology (PUCIT), University of the Punjab (PU), Lahore, Pakistan; 5 Faculty of Information and Communication Technology, Universiti Tunku Abdul Rahman, Kampar, Perak, Malaysia

**Keywords:** Programming, Keyword extraction, Graph-based keyword extraction

## Abstract

Keyword extraction is essential in determining influenced keywords from huge documents as the research repositories are becoming massive in volume day by day. The research community is drowning in data and starving for information. The keywords are the words that describe the theme of the whole document in a precise way by consisting of just a few words. Furthermore, many state-of-the-art approaches are available for keyword extraction from a huge collection of documents and are classified into three types, the statistical approaches, machine learning, and graph-based methods. The machine learning approaches require a large training dataset that needs to be developed manually by domain experts, which sometimes is difficult to produce while determining influenced keywords. However, this research focused on enhancing state-of-the-art graph-based methods to extract keywords when the training dataset is unavailable. This research first converted the handcrafted dataset, collected from impact factor journals into *n*-grams combinations, ranging from unigram to pentagram and also enhanced traditional graph-based approaches. The experiment was conducted on a handcrafted dataset, and all methods were applied on it. Domain experts performed the user study to evaluate the results. The results were observed from every method and were evaluated with the user study using precision, recall and f-measure as evaluation matrices. The results showed that the proposed method (FNG-IE) performed well and scored near the machine learning approaches score.

## Introduction

The rate of increase in research data is recorded massive in recent years, and this massive collection of research data makes the extraction of important information as a difficult task. The research community is drowning in data and starving for information (Amin & Chakraborty, 2018). Therefore, in recent years many approaches were used to extract important information from massive corpus. Moreover, these approaches extracted informative data in a precise way by existing of just few words or phrases. These few words or phrases are called keywords or key phrases that describe the whole document's theme precisely, keywords also contain important research terms and techniques (Ashokkumar, Arunkumar & Don, 2018).

The state of the art approaches available for keyword extraction are classified into statistical approaches, machine learning, and graph-based approaches. The statistical approaches accept data as an individual term and produce the results based on the frequency of that term. The term refers to a single word in a document (Li, 2012). In the state-of-the-art Term Frequency, Inverse Document Frequency (TF-IDF) is the commonly used statistical approach. It works on calculating the term frequency in a single document combined with the frequency of that term in all documents. While the machine learning-based approaches work on the basis of word semantics, the machine learning model is trained with a handcrafted training dataset from which the semantics are to be perceived (Bordoloi & Biswas, 2018). Furthermore, the graph-based approaches work on the basis of graphs, whereas the graph is a combination of nodes connected together with the help of arcs. The nodes are the words in a document and the arc is built on the basis of the relationship between nodes (Bordoloi & Biswas, 2018). Furthermore, once the graph is made then graph-based methods are applied to extract the influenced keywords from huge document collection. The statistical approach TF-IDF is a frequency-based approach. It calculates the frequency of terms and then ranks the terms according to their scores (higher–lower (Zimniewicz, Kurowski & Węglarz, 2018)).

The terms with higher scores are considered as keywords. The TF-IDF works on randomly collecting the words from a document and ranking them from higher to lower just based on their frequencies which makes it difficult to produce quality keywords with better word semantics (Chen et al., 2012). Therefore, the Machine learning approaches were used to better understand the word semantics and to produce quality keywords. The machine learning approaches work on the basis of the training dataset which is a pure handcrafted data set by a domain expert. The training dataset contain the important terms which are extracted manually by human (domain expert) while going through all the documents. The raw data (document collection) is then passed through the machine learning model which further considers some factors to be fulfilled by all terms. The terms which fulfill the criteria are then processed through the word semantics of the training dataset. Furthermore, the terms that successfully passes through the model by fulfilling the word semantics are considered as keywords

The graph-based methods work differently as compared to statistical and machine learning methods. The graph-based methods do not require any training dataset to process results and do not work on just random frequency calculation of words (Hashemi, Dowlatshahi & Nezamabadi-pour, 2020). The graph-based methods work on building the graph of words, the words are considered as nodes and the nodes are linked with arcs. The arcs are made on the basis of relationship among nodes (Biswas, 2019). The graph-based methods works on sequential processing of graphs, the nodes with a higher number of connections in a network are considered the keywords. In the state-of-the-art, the machine learning techniques are used to better determine the influenced keywords from massive text corpora using handcrafted training dataset produced by domain experts. However, an effective method for keyword extraction is required to produce quality results similar to machine learning when the training dataset is not available (Sahingoz et al., 2019). Therefore, this research focused on proposed graph-based approaches in order to produce quality results similar to the machine learning approaches but without using any training dataset. The proposed methodology consisted of some sequential processes starting from the dataset collection and data preprocessing. The data was collected from open access journals related to textile research because this research considered full text articles instead of just abstract and title. The collected articles were preprocessed in order to get only useful words by removing waste data.

The preprocessed data was further converted to *n*-gram combinations ranging from unigrams to pentagrams. The TF-IDF was applied to all *n*-gram combinations and result was observed. The machine learning was also applied to the preprocessed data. Furthermore, to apply graph-based methods all *n*-grams combinations were converted into graphs. The words were considered as nodes and based on co-occurrence of words. The arcs linked the nodes to form graph. The next step is to apply graph-based methods on graph, the methods are PageRank, Hyper Text Induced Topic Search (HITS) and proposed method Frequent Node Graph-based Information Retrieval (FNG-IE) method.

The results from all methods were observed and evaluated based on the user study using precision and recall as evaluation parameters. The user study was performed by domain experts in which the important terms were extracted from all documents. The precision and recall score for every method was observed. Moreover, this research observed that among all *n*-gram combinations, the bigram performed better. The bigram result for all methods was considered to be further compared with the TF-IDF and machine learning approaches result in order to determine the best performing approach. The precision and recall score for HITS-I and the proposed method Frequent Node method were observed near to machine learning method results and much better than the TF-IDF and traditional PageRank Method. However, it was concluded that the proposed approach is the best alternative to machine learning when the training dataset is not available.

## Literature review

The literature study is performed considering the keyword extraction techniques used to extract useful information from huge data collection. The constraints on which the information is considered important and meaningful, the metadata, and the feature set used to process metadata to further extract more useful (required keywords) information. The literature is classified into graph-based, non-graph-based, and machine learning-based methods of keyword extraction, the type of data, quantity, and quality of data, possible outcomes, and limitations of proposed methods. The evaluation parameters for both (graph-based and non-graph-based) methods. A detailed overview of the literature review is given in Table 1.

**Table 1:** Critical analysis of literature studies.

Ref.	Category	Methodology	Datasets	Strengths	Weaknesses
Hong & Zhen (2012), Turney (2003), Khodaei, Shahabi & Li (2012) and Lee et al. (2013)	Statistical methods	Documents are preprocessed and statistical methods are applied to calculate frequencies, and then after frequency-based ranking of words the keywords are prepared TF-IDF and Frequent Pattern method is the most common	Newspaper data Twitter benchmark datasets	Feasible for smaller sized datasets. Best performer in simple structured datasets where only frequency matters	Not feasible for large size and complex structured datasets.
Zhang & Tang (2013), Jain & Gupta (2018), Schluter (2014) and Beliga, Meštrović & Martinčić-Ipšić (2014)	Machine learning	Documents sets are cleaned by preprocessing and are further processed through machine learning methods that work on understanding word semantics from the training dataset. The Quality Phrase Mining approach is state-of-the-art and most common for this purpose	Essays collections Twitter datasets Collection of web data	Does not crash on large size and complex structured datasets	Needs a well developed training dataset
Lahiri, Choudhury & Caragea (2014), Coppola et al. (2019), Zhang et al. (2016), Zhou et al. (2019), Abilhoa & De Castro (2014), Chang, Huang & Lin (2015), Rousseau & Vazirgiannis (2015) and Liu, Chen & Song (2002)	Graph-based	Documents are preprocessed and the feature set is converted into a graph with nodes and edges linkage, then graph-based methods are applied to it. HITS, PageRank, CoreRank, and Centrality measures are the most common and state-of-the-art approaches	News datasets from the web Accidents datasets extraction from web	Supports larger size documents. Works on the basis of nodes and edge connectivity which supports any type of dataset. Does not require training dataset	Limited to graph-based methods

Going through relative literature studies is a vital task to perform research in any specific field of study. It gives a comprehensive overview of research problems, methods and results. However, a huge amount of research done in the textile in past years is difficult to quickly identify the methods, their implementations, and expected outcomes because a research student has to pass through a bundle of research articles of relevant research. To overcome this time-consuming process, data mining methods are used to extract important information. Keyword extraction methods in the data mining process serve the purpose of important keyword extraction from bulks of data to quickly identify important keywords (on given constraints) (Onan, Korukoğlu & Bulut, 2016).

Keyword extraction plays an important part in extracting valuable information from large text corpora, the process varies concerning different text types, but the expected outcomes are always identical. However, a variety of data types, keyword extraction techniques were enhanced, merged with other techniques, and transformed into enhanced ones. The literature study for this research has been clustered regarding techniques to build a quick understanding of those techniques, implementation, and results. The techniques are classified as statistical approaches, machine learning approaches and graph-based approaches.

### Statistical approaches

The keyword extraction methods require a set of features to be selected from the collection of data. The feature set may contain a specific part of speech or combination of two or more, it may be noun, verb or combination of both. The feature set is further processed using the keyword extraction method, in terms of statistical approaches, TF-IDF is most common and validated method (Hong & Zhen, 2012).

The initial step is to collect documents. The documents are kept in one specific format to be further processed (i.e., text format or PDF), it depends on the type of data and experiment, which is to be conducted on given constraints. The next step is to calculate term frequency; the term frequency is calculated by identifying a specific term. Every document is of different length, so there is a chance of high and low occurrence. The term frequency is calculated by dividing the total number of times a term occurs in a document with all (total number of) terms in a specific document.

The term frequency is calculated by dividing the total number of times a term occurs in a document with all (total number of) terms in a specific document. The next step is to calculate the inverse document frequency, which is calculated by dividing the total number of documents by the actual number of documents, including the terms. Moreover, after calculating these two scores, the next step is to combine the frequencies to calculate the final score. However, after calculating the scores for all terms, the next and last step is to rank the terms according to the scores.

The different types of datasets and feature sets need enhancements in the traditional TF-IDF approach, in the sense of improvement while overcoming the limitations expressed by Chen et al. (2012) and Turney (2003) that TF-IDF does not work on complex and huge datasets, another research (Lee et al., 2013) was conducted, considering the spatial factor of web data. The research proposed method named as SKIF-P based on spatial TF-IDF in which the traditional technique was improved.

The proposed method was applied to web pages ranking with hybrid index structures to handle point-based indexing of web documents in an efficient number. The proposed method's validity was validated with multiple experiments, and improved results showed that the addition (Spatial-TF-IDF) worked efficiently (Khodaei, Shahabi & Li, 2012). The deep analysis of previous studies, statistical techniques can only work on datasets of small size; when it comes to the processing of large-sized and complex datasets, traditional statistical techniques are deficient in performing results. Also, there exists no specific and sufficient technique that helps in visualizing the results. So, there was the need for such techniques that perform on large datasets and give improved results on small datasets also. Graph-based approaches and the machine learning approaches were used in comparison with non-graph-based (statistical) approaches.

### Machine learning approaches

The statistical keyword extraction techniques are not sufficient in extracting meaningful keywords from huge and complex structured documents (Turney, 2003); another platform has significant importance in almost every technology-related field, that is machine learning. Machine learning also serves to extract keyword extraction from huge amounts of data even with complex structures and noisy inputs. Its greatness is that it runs on training data that verifies the required pattern, and then based on that pattern, it identifies the most relevant keywords from data collection.

The SVM classifier is a common machine learning-based approach trained with the pattern that is to be identified from a massive collection of text. The research (Lee et al., 2013) has used SVM on spoken documents; the first phase was to calculate topic coherence with term significance measure. The research used both negative and positive segments to train the SVM. The dataset results showed better results in the precision score and the second stage effectively processed better keywords.

The KEA algorithm overcame SVM’s deficiency in extracting quality keywords. The research (Zhang & Tang, 2013) used a KEA algorithm, a combination of statistical and Naive Bayes classifier approach; it also needs to be trained with a valid set of arguments that are fundamental to information extracted from massive text collections. The proposed methods consisted of semantic information and KEA based on Reget's thesaurus (Zhang & Tang, 2013). It included semantic similarity between terms was used to construct the lexical chain, and the length of that chain was further used for building the extraction model.

Quality Phrase Mining is a machine learning-based method that needs a training dataset to produce results. Its name is used to extract quality words with the highest understandability and relevancy to the training data. The research (Zhang & Tang, 2013) has applied QPM with minimal training data and phrasal segmentation to achieve quality keywords. The research also briefly describes the factors on which the quality words are extracted from a massive text corpus. The machine learning techniques perform far better than other techniques because these techniques understand the patterns of data given as training dataset; based on training data’s features, it estimates the quality of words from given as an unprocessed dataset. The machine learning techniques perform far better than other techniques because these techniques understand the patterns of data given as training dataset; based on training data’s features, to be compared with terms in raw dataset.

It starts with the preprocessing of datasets that are composed of documents. The next phase is identifying phrases that are frequently appearing in the document(s), then it goes to the next step that is the assessment of the quality of phrases. The quality of phrases is estimated based on some factors such as understandability, informativeness, and frequent occurrence. The factors vary from research to research because of different requirements and also due to diverse data types. The words that fulfill all the mentioned criteria by passing through all the factors responsible for estimating the quality are stored for the next process (Jain & Gupta, 2018).

The successful phrase segmentation leads to the next step, which is rectifying the frequency of phrases (if needed), and after this last step, the quality phrases are generated (Sahingoz et al., 2019). Furthermore, various approaches were determined in the literature survey; the approaches were carefully analyzed in terms of implication, how they were applied to various types of datasets, and the results after the implementation. The techniques were classified into three basic classes, the statistical ones, graph-based, and machine learning. It is concluded that the graph-based techniques performed relatively best compared to the statistical ones, so this research study considered the graph-based methods to be applied to determine the research trend by using important keywords.

### Graph-based approaches

The datasets are converted into graphed networks based on the selected feature set as explained in the “Research Methodology”, the words are considered nodes, and nodes are combined with the help of edges that are sometimes called arcs; this is called a graph. The techniques that work on graphs are considered as graph-based techniques. The nodes in graph-based datasets are made based on words that are selected as features for further processing, and the edge (or arc) combines two or more nodes to make networks; the arc is made based on constraints that combines the nodes, the common constraints in previous studies are co-occurrence, ranking value.

The graph is composed of nodes and arcs combination; therefore, it supports large and complex datasets to be processed because a graph has a definite pattern. The research (Schluter, 2014) proposed a technique based on centrality measures; it was claimed that by identifying each node’s centrality score and then ranking them based on the highest score, the node could be said as the most important node (node refers to a featured word). The HITS algorithm was used for processing the selected featured graph network. HITS algorithm consists of two scores, hubs and authorities, hubs are the outdegree of a specific node (number of out links pointing to other nodes), and authorities are the in-degree of a specific node (number of links pointing a specific node).

Improvement of understandability of keywords in previously proposed research (Schluter, 2014) based on traditional methods, the research’s (Beliga, Meštrović & Martinčić-Ipšić, 2014) proposed a graphed method that produced better results. According to the previous studies, the research used a graph-based method in the research, this is different and comparatively improved. Instead of traditional HITS and PageRank, this research used a new parameter for the extraction of keywords. The parameter used in the research is the selectivity of nodes. The research defines the selectivity as the average weight on links of a single edge node. The research (Beliga, Meštrović & Martinčić-Ipšić, 2014) proposed the study on the HINA dataset to get results. The increase in F1 and F2 score by approx. A total of 3 percent concerning previous performance proves the improvement of the proposed method.

The graph network comprises nodes, and edges are used to combine nodes based on given constraints as given in the “Research Methodology”. The recent graph-based research was improved in the research (Lahiri, Choudhury & Caragea, 2014) by making a few enhancements in choosing the feature set and using another parameter than the previous one. The research used centrality measures to extract keywords instead of previous PageRank and HITS. The experiment was performed on some benchmark datasets. The proposed method performed well and improved the results as compared to the previous ones. Still, in some places, it was not better performing at all, however, the average result of F-measure was relatively improved as compared to other approaches.

(1) }{}G = V,E

It works on calculating the popularity by determining the visitor nodes to a specific node. To understand how the PageRank score is computed using nodes and links, let’s consider a graph given formula in the Eq. (1) with nodes as V and edges as E. The Eq. (2) below can be used to determine the implementable process of the PageRank algorithm (Coppola et al., 2019).

(2) }{}{\rm PR}\left( {\rm s} \right) = {\rm \; }\mathop \sum \limits_{v\epsilon Bs} \displaystyle{{R\left( v \right)} \over {Nv}}\; \; \; \; \; \; \; \; \; \; \; \; \; \; \; \; \; \; \; \; \; \; \; \; \; \; \; \; \; \; \; \; \; \; \; \; \; \; \; \; \; \; \; \;

The PR(s) represents the PageRank of any node s belonging to Nodes V, where Bs is the collection of all those nodes which are pointing to s, Nv represents the number of the edges going out of the node s. The Rv is the PageRank of node v. Equation (2) shows the PageRank concept implemented to graph-based networks (Zhang et al., 2016; Zhou et al., 2019).

The research was performed in the improvement of PageRank with other traditional graph-based techniques. The research (Abilhoa & De Castro, 2014) overcomes the limitation of VSM (Vector Space Model) for keyword extracts such as sparsity and scalability. The author named his proposed approach as TKG (Twitter Keyword Graph), which uses centrality measures to extract keywords. It used the tweets' data and the represents texts of the tweets as nodes of the graph. The research performed three experiments; in the first experiment, the technique TKG was compared with the literature techniques, and the second experiment was compared with the traditional techniques of TF-IDF and KEA (Keyword Extraction Algorithm). In all three experiments, the proposed approach performed higher robustness as compared to traditional techniques.

To improve keyword extraction results, the research (Chang, Huang & Lin, 2015) proposed a new parameter towards the enhancement of previously described keyword extraction. The research used a feature set based on the co-occurrence of words in a graph. The proposed method used the TF-IDF approach and build three graphs, word to word, sentence to sentence, and sentence to word, and used co-occurrence importance of words to perform keyword extraction using centrality measures. The results assured the improvement of the method.

The previous graph-based techniques were based on centrality measures with different feature sets for extracting keywords. The research (Rousseau & Vazirgiannis, 2015) applied the *k*-core method to represent the document as a graph of words. It retains nodes from the only central core for representing terms. The proposed approach performs better in proximity between the keywords and the variability in the number of keywords extracted through subsets of nodes. The results showed a remarkable improvement in the curves of precision, recall and F-measure as evaluation parameters.

Statistical methods perform keyword extraction but are not sufficient for complex documents or their performance becomes low in dealing with huge datasets. The reason is that graph-based methods are used, but these methods also face scalability issues. To cope with scalability and sparsity limitations, the research (Zhang et al., 2018) proposed a weighted graph-based approach named Keyword from Weighted Graph (KWG). KWG is an unsupervised approach that uses node edge to rank the nodes and centrality measures to calculate the node’s neighborhood closeness and importance. The research applied the proposed scheme on the dataset of the American election and URI attack. The results were comparatively improved, with reasonable enhancement in the results of graph-based techniques, there still exists a deficiency in acquiring the means required results. The literature studies concluded that the statistical approaches lack in providing quality information from huge data repositories but graph-based approaches are far better than statistical ones. The machine learning approaches perform better than all but need training dataset to process data. There exists a need for an approach to produce results such as machine learning approaches results but without training dataset.

## Research methodology

**Figure 1:**
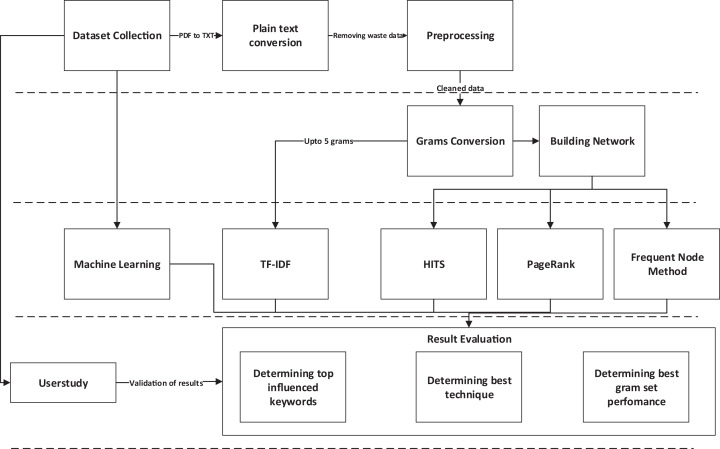
Research methodology.

The proposed research methodology consists of five major phases, which further consists of some steps to be executed sequentially. The first phase consists of data collection. The second phase consists of a set of techniques used to clean the dataset by removing useless words or characters. The third phase comprises *n*-grams generation and building the network of graphs to prepare data to be used for graph-based techniques. The next, fourth phase deals with applying all state-of-the-art techniques on previously scheduled data. The fifth and last phase of methodology comprises of some steps used to validate results and finalize the results; the quick view of research methodology can be perceived from Fig. 1.

### Data collection and plain text conversion

This research tried many benchmark datasets, but no such dataset fulfilled the requirement. So, this research focused on creating a dataset on its own by collecting articles that are closely related to the textile domain and its relevant sub-domains (without considering any specific domain). This research considered full-text articles and the articles were collected from open access impact factor journals because subscription-based journals only provide abstracts and titles without a subscription. Further, only English language articles were selected to avoid any language barrier. The number of articles was nearly 1,000, collected from the year 2015–2019 (a span of 5 recent years). The number of articles were chosen 1,000 for experimentation purpose, can be enhanced in future perspective of this research. The most common sources were Sage Publications, Science direct, Springer and MDPI. The collected data was further converted to plain text that is acceptable for the format of the processing. The initial format of the data was in portable document format (.pdf) format and after plain text conversion it was converted into text format (.txt).

### Preprocessing

The data after plain text conversion was further cleaned by performing preprocessing on it to remove useless words or characters from sentences. The preprocessing step includes tokenization in which the whole sentence is converted into every single tokens, the stop words removal, which is responsible of removing useless words (usually concatenation words or symbols). The stemming deals with the conversion of every word to its root word, the stemming was chosen over lemmatization because this research added a constraint in stemming process in which the words ending with “ing” and “ed” were passed through checkpoint and all other were discarded. At the last the parts of speech tagging which deals with the selection of specific parts of speech. This research chose the nouns and verbs to be filtered while performing parts of speech tagging.

### *N*-grams conversion

When applied for extracting useful information from a huge sized corpus, the data analysis process is always a need for such methods that improve word semantics to improve the understandability of words. There exist many methods, and one of the most commonly used methods in literature studies is *n*-grams. It is a sequence of words arranged so that a word combines with its adjacent word. The “*n*” in *n*-grams means the number of grams, gram is considered a specific word (Hajek, Barushka & Munk, 2020).

The number of grams represents the final gram set; let’s say if the value of *n* is 1 so the gram set on this value be called as a unigram, if the value of *n* is 2, the gram set will be called as bigram, and so on (Bisandu, Prasad & Liman, 2018). It can be easily understood by an example considering the making of bigram of a string (sentence.): “Textile industry contributes a reasonable revenue to the economy of the country” if converted to bigram, it will look like “Textile industry” “industry contributes” “contributes a” “a reasonable” “reasonable revenue” “revenue to” “to the” “the economy” “economy of” “of country”. The *n*-grams converted words provide more sense than simple words. This study considered *n*-grams set ranging from unigram to pentagrams to achieve higher understandability of words depending on the applied gram set. The grams set from unigram to pentagrams were made for each year’s data set, for example: for the dataset of the year 2015, there will be 5 g set and the same way for the year 2016, 2017, 2018 and 2019 respectively.

### Statistical approach

According to the research methodology, when *n*-grams conversion got completed, the next step was to apply the keyword extraction techniques to choose, rank and determine the set of keywords. This study has divided the applied technique into three types according to the nature of techniques such as statistical or non-graph-based techniques, graph-based approaching, and the last one machine learning approaches. In this phase, the statistical techniques will be discussed in terms of their practical implementation. According to the literature studies, TF-IDF was found most commonly implemented technique for keyword extraction that is why this research incorporated this technique in order to extract keywords.

#### Term frequency, inverse document frequency

Among other statistical techniques, the most prominent and common technique was TF-IDF (Li, 2012). The TF-IDF is an unsupervised approach which is used to find the frequency of occurrence of any term or in simple a word in a document in ratio to its frequency in the other documents and by combining the frequencies, the final score becomes ready. The terms are ranked further according to their scores. This research also performed TF-IDF on the data filtered out after preprocessing. The final terms ranked in descending order and the top ones were selected.

### Machine learning approach

Implementing machine learning to determine the importance or influenced keywords is also well known and applied in many studies. Following this research strategy, machine learning contains the most common and recommended Quality Phrase Mining (QPM) that works on multiple constraints to filter and rank keyword or key phrases (Zhang & Tang, 2013).

#### Quality phrase mining

The quality phrase mining is a machine learning-based approach that comprises of multiple processes that need to be executed in a particular sequence to get quality keywords. The major reason for using this technique is that it does not just deal with the occurrences or frequencies; it deals with understanding word semantic meanings. This technique required a training data set based on which it will understand the semantics (of training data) and will filter the provided raw datasets accordingly. The QPM in this research is applied directly to the dataset; it firstly cleaned the data by removing useless words and characters from the dataset. The filtered words were further passed through four major factors that were completeness, frequency, informative and understandability in the next process.

The completeness refers to the whole meaning of terms; the frequency means the repetitive occurrence of words in a document. The informativeness means the terms should relate to any standard meaning. The understandability deals with that property of word which is responsible of having a proper meaning. When passed through these four constraints, the filtered words were claimed to be the best influenced or quality words. The resultant word passed through these four constraints were at last matched with the semantics of the words stored in training datasets. The training dataset was provided to this method, which contained standardized data with the set of semantics expected to be achieved by the method when processed on the raw dataset. Finally, the words which were given by this method were considered the quality words and were recorded in a result file for comparison with other approaches.

To avoid overfitting and underfitting issue, whole dataset was shuffled first, secondly the 60% of data was used to train the model and the rest 40% for testing purpose. Furthermore, to avoid annotators biasedness of annotators, this research chose five annotators directly related to textile field but belonging to different organizations, three out of five were the graduates and the rest two belonged to the industrial practicing field of textile. The data acquired from the annotators were collected, shuffled and saved as a single study and was further divided into training and testing purpose.

### Building graphs

The number of *n*-gram set of each year gives the number of graphs for each year; this research has made 5 g set for a single year dataset (ranging from unigram to pentagrams). The gram set result is saved in the form of a list in text files (for each gram set unigram to pentagram). The graph consists of two main things, the nodes and the arcs. In the context of this research, the nodes are the words that are saved in the form of gram sets (unigram–pentagram).

The nodes are combined with arcs’ help, and arcs are made between nodes based on co-occurrence (Zhou et al., 2019). The graph generation started with the assigning of node number to the nodes existing in the files (list of gram sets in the files). However, by carefully keeping in view that no separate number should be assigned to the same node (word) existing in any other location, if the word existed on any other location it was assigned that number which was assigned previously. For example: if node number of node “quality” is “55” and on “l00” location it appears again then it will not be assigned “100” to “quality” it will be recognized by the previous node number that is “55”. The graph of words network once made, was further used to perform graph based analysis.

### Graph-based approaches

The literature studies consisted of many approaches and were applied according to the data and the required result. This research selected those approaches that were better in performance and were most commonly used (according to literature studies) (Coppola et al., 2019). The approaches were HITS and PageRank approach; another reason to select these approaches was the high relevancy of their outcome with the requirement of this study's expectations which was to determine the influenced node from huge collections.

#### Hyper text induced topic search

According to the literature studies, the one of most common graph-based keyword extraction technique is HITS; it works in such a way that firstly, it recognizes the nodes and the structure (the way nodes are connected) of arcs and nodes. The “Graph-Based Approaches” describes the node-edge connectivity in detail. The HITS approach in the context of this research was altered according to the requirements. Just a single iteration of authorities was considered for every node. Hubs are the number of nodes that a specific node is pointing to (outline), and authority is the number of nodes that are pointing that specific node (In links). The number of hubs of a specific node tells us how often a node is connected to other nodes. The number of authorities of a specific node tells us how a node is important in the network as it tells us how many nodes are visited that specific node (Liu, Chen & Song, 2002).

It accepted the network in the form of an input file of .csv file format; the complete network gets completed with two input files. One file contains the complete information about the nodes, such as node number and node name; the second input file contains the connections/links that form the actual network, such as the source node and the target node. The structure of nodes files consists of nodes number and names, which consists of two columns, the first column contained node number and with the heading of “ID” and the second column contained the name of the node with the heading “Name”, the brief overview of its structure can be seen in Table 2.

**Table 2:** Node name file structure.

ID	Name
1	Fiber
2	Construct
3	Yarn
4	Nanoparticle

Similarly, the second file that contains the information of arcs or simply the links by which the network is formed, the connectivity of the source node with the target node. This file also contained two columns; the first column contained the source node number, and its heading was “Source” the second column contained the target node number, and its heading was “Target”. The input files were processed according to their structure; in the first phase, the arcs file was read. The HITS algorithm’s practical implementation was performed by accessing the source and target node numbers (for each specific node), the hubs and authorities were calculated for every node. The calculation was then ranked in highest to lowest order.

This research applied just a single iteration by using just determining each node’s authority scores and ranking it in descending because it has to determine the nodes with the highest authority values. After all, it described the node, which is more influential among other nodes (Sanchez et al., 2019).

The second phase was to print the names of nodes from the second file, the node numbers of highly ranked nodes were matched from the nodes name files, and hence the knowledgeable results were made when the names of influenced nodes were printed. The top nodes with the highest scores were printed for further use. Python was the language used to implement the above methodology, the NumPy, matplotlib, sklearn, and networkx libraries were used to compute various calculations within the algorithm; these techniques were validated to be used in many literature studies.

#### PageRank

The PageRank approach processes likely to HITS algorithm, a commonly used graph-based method following the literature studies. The PageRank method also works to determine important nodes. PageRank is defined as a method among all centrality measures that work on the centrality of nodes pointing to a specific node and centrality of the nodes that are pointing to those nodes. The process continues till the final calculation until the whole graph is traversed.

The input files were prepared in the same way as HITS and were accessed in the same way as HITS accessed the source and target nodes. The Table 2 represents the file structure for nodes, and Table 3 shows the structure of the input file for arcs. The PageRank was calculated firstly from the arcs file where there were source and target nodes, the centrality of all nodes pointing to a specific node, and the centrality of a node, which is pointed by all other nodes. The output or expected results and the methodology is similar to HITS to some extent. Still, in practical implementation and modeling, both are different and as well as different in structure. The produced results of PageRank were influenced terms ranked in higher to lower scores (shown in “Results”).

**Table 3:** Arcs file structure.

Source	Target
1	2
2	3
3	4
4	5

#### FNG-IE approach

The FNG-IE method is based on the concept of statistical method, known as a frequent pattern method. The working concept of this method relates to the frequent pattern method but is enhanced in such a way that it works on graph-based dataset. The graph structure was firstly read by the proposed method and then the node structure was accessed to be executed. Furthermore, this method calculates the frequency of nodes and then ranks the nodes according to their scores. Firstly, it identifies the nodes, the source node, and the target node; it considers the calculation of frequencies for both nodes and then finds its ratio with the total number of nodes, hence the final score calculated for each node. Moreover, the node score for each node is then matched with the node name, which is saved in the nodes file. The node names are printed then as highly influenced nodes.

**Figure 2:**
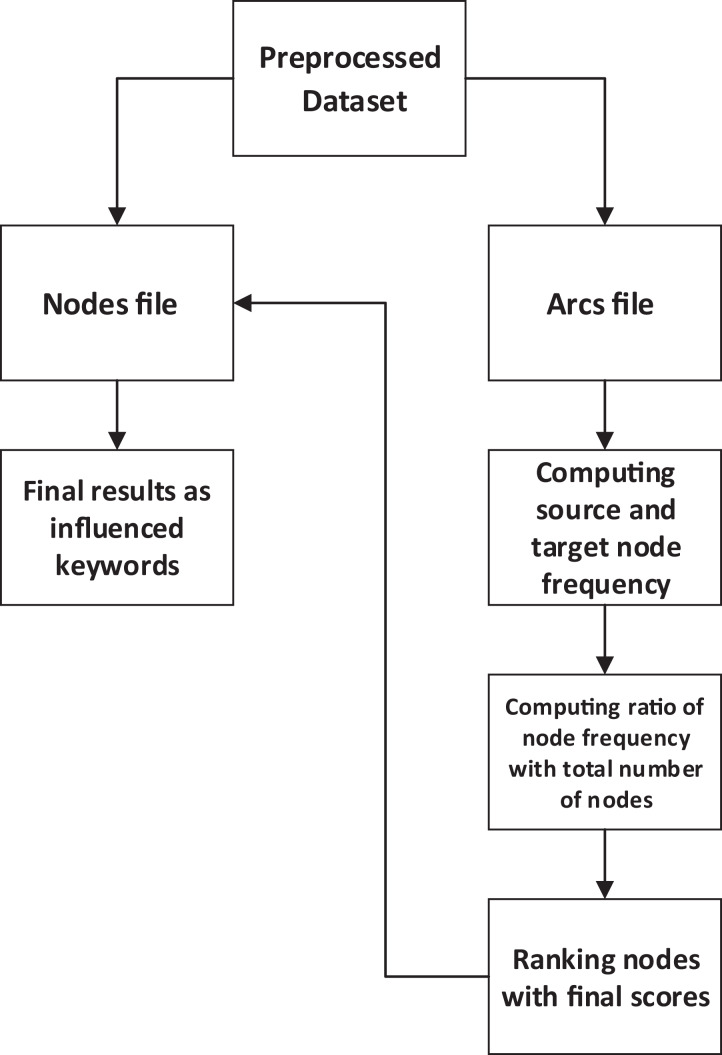
FNG-IE methods conceptual overview.

The preprocessed terms were converted into graphs, as described in the “Hyper Text Induced Topic Search”. The two files were made of which one is called as nodes file, and the second was the arcs file. The nodes file contained the term and the number of its nodes and the arcs file containing the source node and target node description for each node. The proposed method is given in the Fig. 2 that reads the linkage mechanism of arcs from the arcs file, and for each node, it computes the frequency for both target and source node because just by calculating the frequency of only target nodes does not give the complete influence of a node in a network. Although the target node is important because it is being hit by other nodes but the importance of source node cannot be avoided in such a case when the influence node is required. This research’s absolute target was determining the influence node, so source node was also considered as source node was also in repetitive occurrence in many places (within a network).

Moreover, when the frequency is calculated, the next step is to figure out the ratio of a specific node’s score to the total number of nodes in a network. Moreover, after this computation, every node has a final score that is further used to rank the nodes according to their score (from a higher score to lower score). The final score is stored against each node, and then the node number is matched with the file containing node number and the node name; this was done to print the node names because the end results need to be understandable. These node names are words described in “Hyper Text Induced Topic Search” and these words after the whole processing are considered to be the important or influenced keywords.

## Results

This research has considered precision, recall and f-measure as evaluation parameters, these parameters were selected keeping in view the literature studies. Precision is considered an evaluation parameter in terms of information retrieval, according to the literature studies. Precision works based on relevant information (or data) and retrieved information (or data). The Eq. (3) refers to the mathematical overview of Precision.

(3) \rm Precision{\rm \; }score = \displaystyle{{True{\rm \; }Positives} \over {True{\rm \; }Positives{\rm \; } + False{\rm \; }Positives}}{\rm \; \; \; \; \; \; \; \; \; \; \; \; \; \; \; \; \; \; \; \; \; \; }

The terms true positive refers to the results containing the data present in the actual document and the predicted document. The False-positive refers to the results which contain the data which is not present in the actual document but is predicted by the experimentation (Arora, Kanjilal & Varshney, 2016). The recall is the ratio of true positives over predicted results and can be determined by the following formula given in the Eq. (4).

(4) \rm Recall{\rm \; }score = {\rm \; }\displaystyle{{True{\rm \; }Positive} \over {True{\rm \; }Positive + False{\rm \; }Negative}}{\rm \; \; \; \; \; \; \; \; \; \; \; \; \; \; \; \; \; \; \; \; \; \; \; \; \; \; \; \; \; }

The recall score indicates the percentage of the total number of relevant results that are accurately identified by the method. The terms true positive and false negative are discussed in “Results”. The recall score usually turns less than the precision. Still, this condition is not obvious in all cases, recall score increases in many cases where the algorithm performs well and observes the better results (Batalha et al., 2019). The f-measure is also calculated among other evaluation parameters (precision and recall). The f-measure is basically the harmonic mean that is calculated from the scores of precision and recall as shown in Eq. (5).

(5) }{}F - \rm Measure{\rm \; } = {\rm \; }2\displaystyle{{Precision{\rm \; } \times Recall} \over {Precision + Recall}}{\rm \; \; \; \; \; \; \; \; \; \; \; \; \; \; \; \; \; \; \; \; \; \; \; \; \; \; \; \; \; \; \; \; \; \; \; \; \; \; \; \; \; \; \; \; \; \; \; \; }

This research has considered the calculation of these parameters for result evaluation. In literature studies, it is closely observed that the most common evaluation parameters are recall, precision and the *F*-measure score.

### *N*-gram based result

This research classified the results in the combination of *n*-grams, from unigram to pentagrams combination. The statistics of the results are shown in the form of graphs in the subsequent Section. The results are arranged in a way that for each *n*-grams combination, every statistical and graph-based method was applied and the result was observed. In the same way, the results are shown after evaluating by the evaluation parameters discussed in the “Results”. The graphs show the curve of evaluation score ranging from 0 to 1, having a different score for every different method. The higher precision, recall and *F*-measure score on the graph shows the better performance of the method.

#### Unigram result

**Figure 3:**
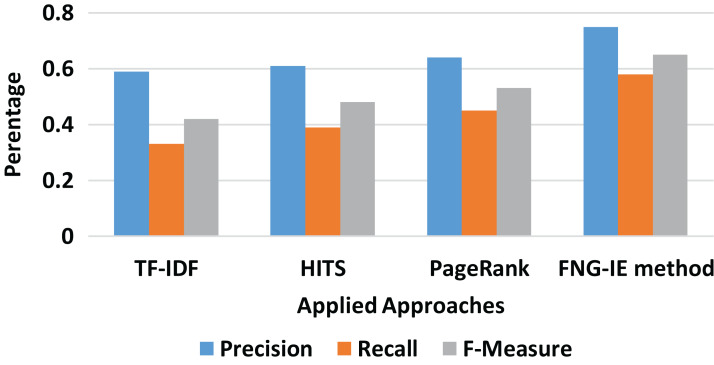
Unigram results of all methods.

It can be clearly seen from Fig. 3 that on the unigram dataset, the TF-IDF approach performed relatively less as compared to the graph-based methods. Moreover, in terms of graph-based methods, the HITS and PageRank performed nearly similar results, but the FNG-IE method performed better than all other methods as its score of precision and recall is higher than all other methods.

#### Bigram result

**Figure 4:**
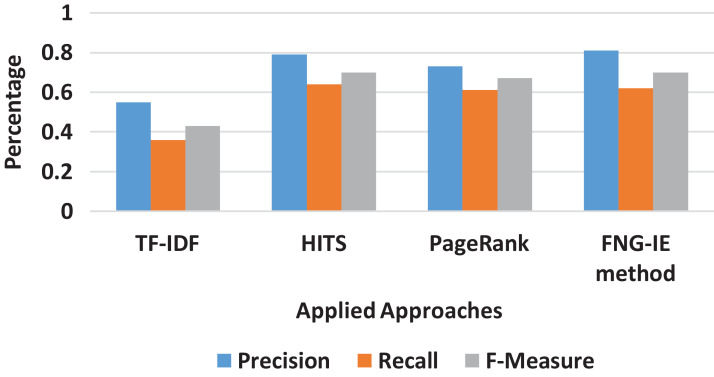
Bigram results of all methods.

The bigram converted dataset consisted of two neighboring words (terms) as described in “*N*-Grams Conversion” was also processed the same way as the previous “Results” all the methods were applied to the bigram dataset. The results were evaluated with the calculation of precision and recall score, and Fig. 4 shows the curves of scores ranging from 0 to 1. The results show that the TF-IDF as statistical method scores are again less than that of graph ones and in graph ones, the FNG-IE method performed well as compared to other graph-based methods. The HITS and the PageRank are again close, but the HITS is relatively better than the PageRank. The factor to focus on the results of bigrams is that it scored neighbored the score of the machine learning method, and the bigram dataset performed well as compared to other *n*-grams methods.

#### Trigram result

**Figure 5:**
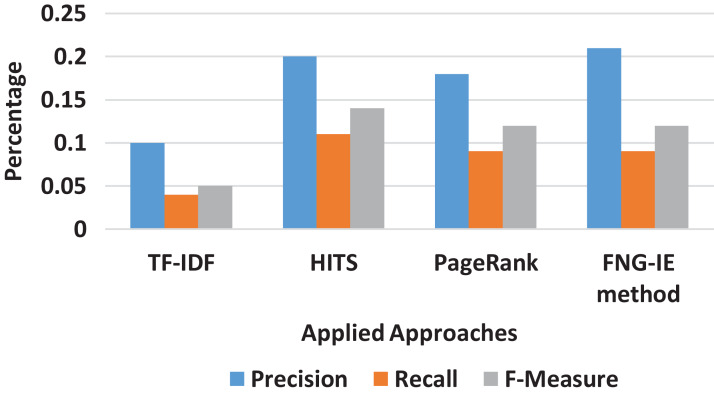
Trigram results of all methods.

The trigram converted dataset consisted of three neighboring words (terms) was also processed the same way as the previous “Results” all selected methods of this research’s methodology were applied to the trigram dataset. The results were evaluated with the calculation of precision and recall score, and Fig. 5 shows the curves of scores ranging from minimum 0 to the maximum 1. The results show that the TF-IDF as statistical method scores are again less than that of graph ones, and in graph ones, the FNG-IE method performed well compared to other graph-based methods. The HITS and the PageRank are again close, but the HITS is relatively better than the PageRank. The most important thing to consider here is that the maximum outcome of precision and recall score is near 0.2, which means that the trigrams are less likely to become influenced keywords.

#### Four gram result

**Figure 6:**
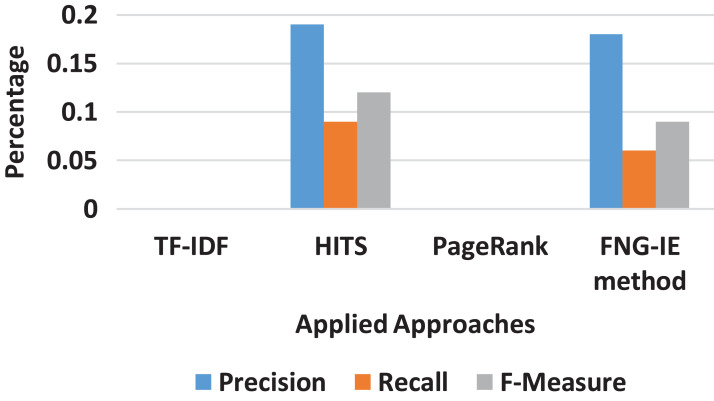
Four gram results of all methods.

The dataset of four gram was next processed with all methods as described in “Results”. The results were recorded in the form of curves that ranges from 0 to 1. The four gram results were also near to trigram, but the recall score was relatively low that it ranges under 0.1 range. The result means that the four gram method does not produce important keywords in a huge number. Figure 6 shows the statistics in the form curves, whereas in the case of pentagrams, where the *n*-grams set was the combination of five consecutive words got the precision and recall core of 0. Furthermore, the graphed display of pentagram was not effective to display.

#### Machine learning result

**Figure 7:**
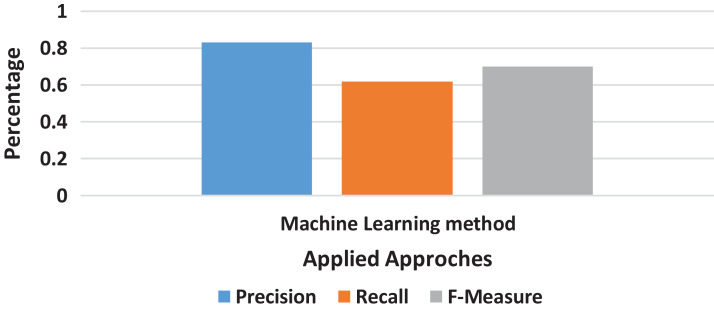
Machine Learning method result.

The machine learning method does not relate to all other methods used in this research. It totally differs in structure, the way of processing, and the display of results also. The very first thing as compared to other methods is that it does not work on the basis of *n*-grams because it accepts the whole document and provides results on quality phrase mining’s method as described as in Fig. 6; the precision and recall score calculated on the results from machine learning methods were relatively better than the scores of all other methods given in Fig. 7.

The machine learning method’s performance was observed better than all *n*-grams converted dataset from all the methods chosen by this research. The precision and recall scores were recorded for all methods in an effective manner.

### Overall result comparison

**Figure 8:**
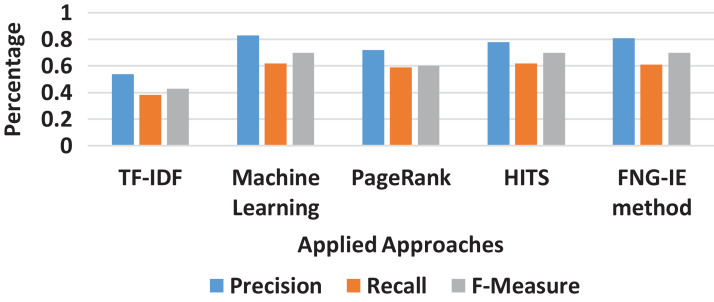
Overall statistics of all applied approaches.

It is also observed that among all *n*-grams combination, the dataset with bigram performed the best precision and recalled with all the methods applied. The bigram dataset’s precision and recall result curves approached almost similar statistics to the machine learning method. Furthermore, among all other graph-based methods FNG-IE method was proposed by this research. The standard methods, HITS, and PageRank performed better than the statistical methods but HITS methods performed better than the traditional PageRank method after enhancement by this research. The FNG-IE method's statistics and enhanced HITS can be seen as better performers in graphs of all *n*-grams combinations. Furthermore, the bigram among all *n*-grams performed better than others. The best performer in all *n*-grams combinations, the bigram results of all methods were comparatively measured with the results of the machine learning method. The overall statistics of this research are given in Fig. 8.

The results showed that the random methods, such as TF-IDF which works on just a single factor that is frequency, is not capable of dealing with the huge sized dataset. The statistics of precision and recall scores of TF-IDF can be seen in “Results” and further in Figs. 3–8 showing overall statistics of all methods of all datasets. Moreover, it proves that only a single factor is not enough to deal with huge datasets. This is the reason this research considered *n*-gram combinations, to make data more informative. Furthermore, the machine learning method performed better than all methods because it's processing is based on a training dataset that makes it strong to extract quality words. However, the proposed method was based on graph connectivity and was enhanced in such a way that it works without any training dataset and has produced better results than the traditional PageRank method. The results produced by th proposed methods were better than the statistical and the traditional graph-based method because it was enhanced by minimizing the limitations and considering the all-important factors that are essential in producing good results. The Table 4 shows the comparative statistics of all methods.

**Table 4:** Comparison of results.

Methods	Precision	Recall	*F*-measure
TF-IDF	0.54	0.38	0.43
Machine learning	0.83	0.62	0.7
PageRank	0.72	0.59	0.6
HITS-I	0.78	0.62	0.7
FNG-IE method	0.81	0.61	0.7

Furthermore, selecting only nouns and verbs from the dataset as described in “Preprocessing” proved the reason behind the better results of the proposed approach. The selection of nouns and verbs did the job of determining the meaningful and important words from whole documents. The enhanced methods described in “Graph-Based Approaches” and were performed on the filtered words in the form of graphs. The results validated the proposed method as the best performers among all state of the art approaches used in this research.

## Conclusion

The machine learning methods are state-of-the-art methods in determining the keywords from a huge collection of the document but require the training dataset. The training dataset is made by domain experts manually by assessing the collection of documents thoroughly. In the situations where the training dataset is not available, machine learning approaches are not applicable. However, The statistical techniques as described above, are not sufficient to deal with huge datasets, so this research works in a different manner than machine learning by focusing on enhancing state-of-the-art graph-based approaches to perform better in situations where the training dataset is not possible to collect. This research used *n*-grams combinations of the handcrafted dataset, which was collected from the journals of the textile domain. Furthermore, the graph-based approaches were further enhanced by adding constraints according to the required results. The methods were applied to all *n*-grams converted datasets, and the best *n*-grams combination was determined as bigram because it produced the best results, among others. Moreover, for evaluation purposes, the user study was conducted with the help of textile domain experts that were graduates of textile field. The results from all methods were analyzed with user study using precision, recall and f-measure scores. This research’s proposed approach is a novel method to extract keywords with comparable quality of machine learning methods but without any specific training dataset. The proposed method scored 4 percent behind the machine learning approach and 30 percent ahead of the statistical approach; this concludes that the proposed approach is the best alternative in situations where a large training dataset is not available.

## Supplemental Information

10.7717/peerj-cs.389/supp-1 Supplemental Information 1 Code files.

## Additional Information and Declarations

### Data Availability

The following information was supplied regarding data availability: Code is available in the Supplemental Files.
